# Relationship between frailty and mortality, hospitalization, and cardiovascular diseases in diabetes: a systematic review and meta-analysis

**DOI:** 10.1186/s12933-019-0885-2

**Published:** 2019-06-18

**Authors:** Satoshi Ida, Ryutaro Kaneko, Kanako Imataka, Kazuya Murata

**Affiliations:** Department of Diabetes and Metabolism, Ise Red Cross Hospital, 1-471-2, Funae, 1-chome, Ise-shi, Mie 516-8512 Japan

**Keywords:** Comorbidities, Elderly, Frailty, Mortality, Meta-analysis, Cardiovascular disease, Hospitalization, Observational study

## Abstract

**Background:**

In patients with diabetes, death and cardiovascular diseases are attributed to classical risk factors such as hypertension, dyslipidemia, and smoking habit, whereas these events are attributed to frailty in the remaining patients. In this meta-analysis, we examined the relationship between frailty and mortality, hospitalization, and cardiovascular diseases in patients with diabetes.

**Methods:**

Literature search was conducted using databases such as MEDLINE, Cochrane Controlled Trials Registry, and ClinicalTrials.gov. Studies that examined the relationship between frailty and mortality, hospitalization, and cardiovascular disease and included hazard ratios (HRs), odds ratios (ORs), and 95% confidence intervals (CIs) were included. Statistical analysis was performed using a random effects model, and pooled HRs, pooled ORs, and 95% CIs were calculated.

**Results:**

The literature search extracted 8 studies (565,039 patients) that met our inclusion criteria, which were included in this meta-analysis. The pooled HR of prefrailty and frailty related to mortality was 1.09 (95% CI 1.01–1.17; P = 0.02) and 1.35 (95% CI 1.05–1.74; P = 0.02), respectively, indicating a significant relationship between them. The pooled OR of prefrailty and frailty related to hospitalization was 2.15 (95% CI 1.30–3.54; P = 0.003) and 5.18 (95% CI 2.68–9.99; P < 0.001), respectively, indicating a significant relationship. Although a significant relationship was found between frailty and cardiovascular diseases, we found only few related studies; thus, robust results could not be obtained.

**Conclusions:**

In patients with diabetes, a significant relationship was observed between frailty and mortality and hospitalization. However, only few heterogeneous studies were included, warranting further examination.

## Background

The number of patients with diabetes is increasing and is expected to reach 300 million globally by 2025 [1, 2]. The primary aim of diabetes treatment is to prevent vascular complications and maintain quality of life (QOL) [3]. Moreover, this treatment is extremely important to improve prognosis and prevent the onset of cardiovascular diseases [4]. Furthermore, hospitalization of patients with diabetes due to complications and severe hypoglycemia has subsequently increased medical expenses [5, 6], indicating that controlling medical expenses related to hospitalization will become increasingly crucial in the future.

Recently, frailty has gained attention in the field of diabetology. Frailty is defined as a condition in which physical and mental activities reduce with age, leading to physical and mental weakness; however, in frailty, activities of daily living and QOL can be maintained through appropriate intervention [7, 8]. In the literature, varying incidence of frailty in middle-aged to elderly patients with diabetes has been reported ranging from 32 to 48% [9]. The prevalence of frailty among community-dwelling elderly is 5–10% [9–11]. In patients with diabetes, chronic inflammation, increased oxidative stress, and insulin resistance cause loss of musculoskeletal mass and muscle weakness, which may increase the incidence of frailty [9, 12]. Furthermore, it is thought that frailty causes chronic inflammation and insulin resistance, which are believed to be closely related to vascular complications and mortality [13]. In patients with diabetes, death and cardiovascular diseases are attributed to classical risk factors such as hypertension, dyslipidemia, and smoking habit in approximately 60% of the patients [14], whereas these events are attributed to frailty in the remaining patients [14–16]. Moreover, frailty is reportedly associated with hospitalization and higher medical expenses [17, 18], which is considered a problem in terms of medical economics. Frailty is thought to improve with appropriate intervention [19–21]; thus, its early detection for early intervention is considered important.

As mentioned above, examination of the relationship of frailty with mortality, hospitalization, and cardiovascular diseases in patients with diabetes is important both clinically and in terms of medical economics. Therefore, the aim of the present study was to comprehensively analyze the relationship between frailty and mortality, hospitalization, and cardiovascular diseases in patients with diabetes through a systematic review and meta-analysis.

## Methods

### Study selection

On December 1, 2018, we conducted literature search using these databases: MEDLINE (from 1960), Cochrane Controlled Trials Registry (from 1960), and ClinicalTrials.gov. In addition, a manual search was conducted on Google Scholar. The literature was searched using the following keywords: [(“Diabetes Mellitus”[Mesh] or diabet*) and (“Frailty”[Mesh] or frail* or “Frail Elderly”[Mesh] or frail elderly) and (“Mortality”[Mesh] or mortality or “Death”[Mesh] or death or “Survival”[Mesh] or survival or “Hospitalization”[Mesh] or hospital* or utilization or “Cardiovascular Diseases”[Mesh] or cardiovascular diseases or “Stroke”[Mesh] or stroke or “Myocardial Infarction”[Mesh] or myocardial infarction or “Angina Pectoris”[Mesh] or angina pectoris or “Coronary Disease”[Mesh] or coronary artery disease or coronary heart disease or “Acute Coronary Syndrome”[Mesh] or acute coronary syndrome or “Heart Failure”[Mesh] or heart failure or cerebrovascular events)]. The studies that examined the relationship between frailty and mortality, hospitalization, and cardiovascular diseases (i.e., myocardial infarction, angina pectoris, heart failure, and stroke), for which hazard ratios (HRs), odds ratios (ORs), and 95% confidence intervals (CIs) could be extracted, were included. Reports such as reviews, letters and commentaries, reports of animal experiments, and overlapping reports were excluded.

When several groups were classified according to the severity of frailty, the severest group was defined as the frail group, whereas the other groups were defined as prefrail groups and their data were used to compare those of a non-frail group. In an event of reports using the same cohort, we used the data with the longest follow-up period. Furthermore, the search was limited to English literature. When the results or findings of a report was difficult to interpret, the co-authors were consulted (RK, KI, and KM).

### Data extraction and quality assessment

We created a data extraction form listing the following study characteristics to be included in the present study: key author’s name, publication year, study location, study design, sample size, participants’ basic information, frailty measurement method, outcome, follow-up period, effect measure, and adjustment factors. Continuous variables are presented as means, standard deviations, standard errors, or 95% CIs, and binary variables are presented as rates (%). If a report included several HRs and ORs, we used the results that were most adjusted for the confounding factors. Quality was evaluated using the Risk of Bias Assessment tool for Non-randomized Studies [22]. Six domains, i.e., participant selection, confounding variables, exposure measurement, blinding of outcome assessors, incomplete outcome date, and selective outcome reporting, were evaluated according to low, moderate, and high risk of bias.

### Statistical analysis

Pooled HRs, pooled ORs, and 95% CIs were calculated for mortality, hospitalization, and cardiovascular diseases associated with frailty. HRs, ORs, and 95% CIs were converted to natural logarithm (logHR), natural logarithm (logOR), and standard error, respectively. Because effect sizes might differ for mortality, hospitalization, and cardiovascular diseases associated with frailty in each study, a random effects model was used for analysis. Statistical heterogeneity was evaluated using *I*^2^ (heterogeneity was determined when *I*^2^ was ≥ 50%) [23]. Subgroup analysis for age (≥ 65 and < 65 years) was performed to examine the relationship between prefrailty/frailty and outcome. If ≥ 10 studies would be included in the analysis, we planned to create a funnel plot to assess publication bias [24]. P-values < 0.05 were considered statistically significant, and analyses were performed using RevMan version 5.3 (Cochrane Collaboration, http://tech.cochrane.org/revman/download; December, 2018).

## Results

### Description of the included studies and assessment of potential bias

**Fig. 1:**
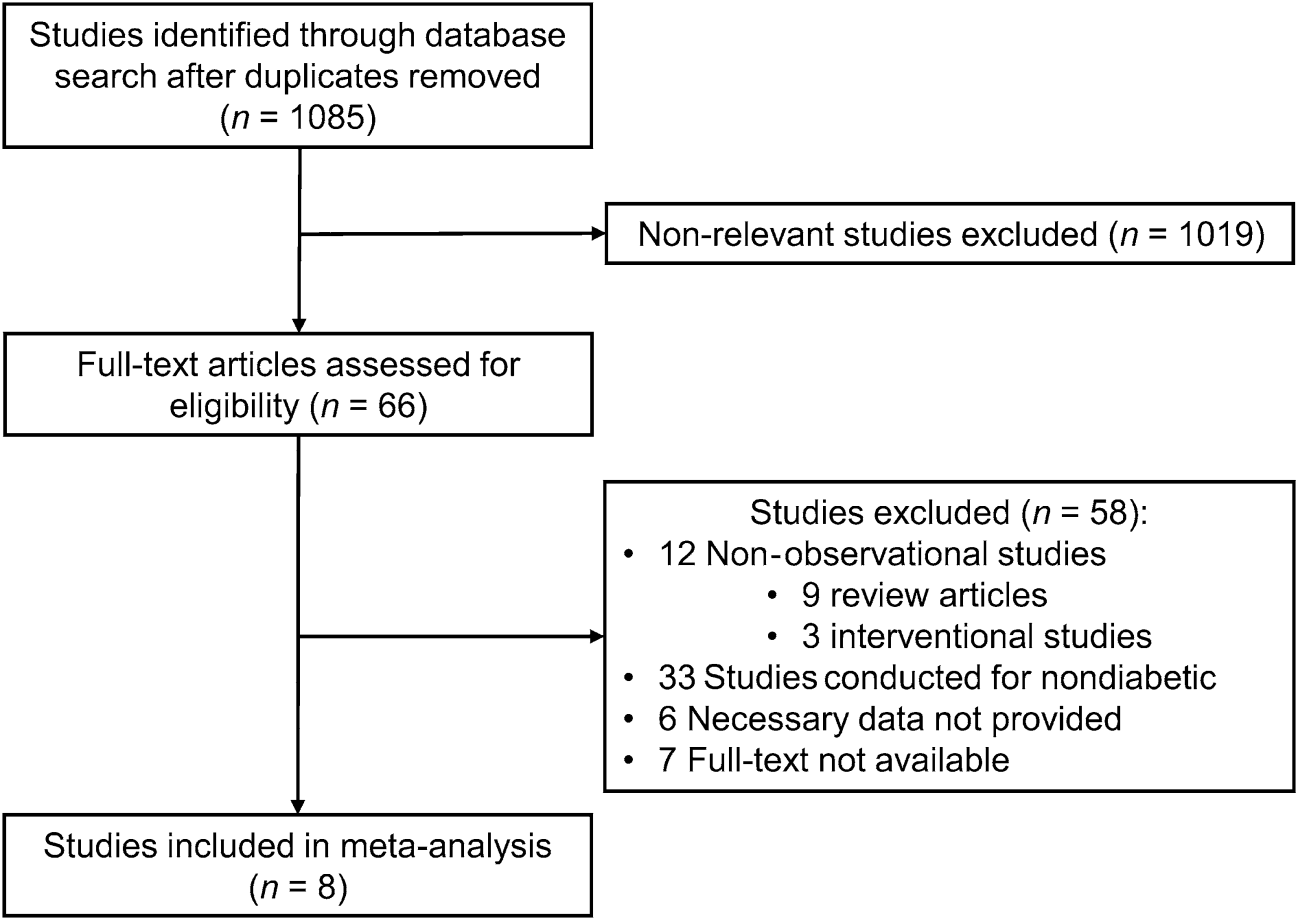
Study flow diagram

Through our literature search, we extracted a total of 1021 reports, among which 8 studies (n = 565,039 patients) met the eligibility criteria and were included in the meta-analysis (Fig. 1) [25–32]. The characteristics of the 8 studies are summarized in Table 1. Of these studies, 7 were longitudinal studies and 1 was a cross-sectional study. The participants included in the sample had a mean age of 68 years, and 53% of them were females. Frailty evaluation was primarily performed using a self-administered questionnaire (the Fatigue, Resistance, Ambulation, Illnesses, and Loss of Weight scale was used in 5 studies], and the incidence of frailty was approximately 24%. The shortest observation period was 0.5 years and longest period was 12 years.

**Table 1:** Characteristics of the studies included in the present meta-analysis

Reference	Year	Region	Design of study	No. of patients	Age (years)	Women (%)	Frailty measurements	Frailty (%)	Outcome	Follow-up period (years)	Effect measure	Adjustment
Cacciatore et al. [25]	2013	Italy	Longitudinal	188	72	67	Frailty staging system	48.4	Mortality	12	HR	Age, sex, BMI, waist circumference, heart rate, pulse blood pressure, Charlson comorbility index, drugs number, GDS, CHF, PAD, and CKD
Wang et al. [26]	2014	US	Longitudinal	2415	73	NR	Marker for a major frailty characteristic	44	Mortality	5.6	HR	Age, race, diabetes duration, age-adjusted Charlson comorbidity score, smoking cessation status, LDL levels, and HbA1c levels
Li et al. [27]	2015	China	Longitudinal	146	80	32	FRAIL scale	15.1	Mortality, hospitalization	2	OR	Age, sex, MMSE points, BMI, duration of diabetes, HbA1c levels, macroangiopathy, and nephropathy
Castro-Rodriguez et al. [28]	2016	Spain	Longitudinal	363	76	54	Rockwood Frailty Index	NR	Mortality	5.5	HR	Age, sex, disability, and cardiovascular disease
Chode et al. [29]	2016	US	Longitudinal	215	57	69	FRAIL scale	NR	Mortality	9	OR	age and sex
Liccini et al. [30]	2016	US	Longitudinal	198	64	47.5	FRAIL scale	28.8	Mortality, hospitalization	0.5	HR; OR	age, sex, education, and HbA1c levels
Chao et al. [31]	2018	Taiwan	Longitudinal	560,795	56	46	FRAIL scale	0.2	Mortality, hospitalization, cardiovascular disease	3	HR	Age, sex, comorbidities (including obesity, mental illnesses, and hypoglycemia history), substance use (smoking and alcohol abuse), aDCSI, and medications
Li et al. [32]	2018	Taiwan	Cross-sectional	719	Aged ≥ 65 years	58	FRAIL scale	9.4	Hospitalization	–	OR	Age, sex, education, marital status, duration of diabetes, use of insulin, falls, ADL disability, and IADL disability

Unless indicated otherwise, data are presented as mean values

HR, hazard ratio; OR, odds ratio; BMI, body mass index; GDS, geriatric depression scale; CHF, chronic heart failure; PAD, peripheral arterial disease; CKD, chronic kidney disease; LDL, low-density lipoprotein; HbA1c, hemoglobin A1c; MMSE, Mini-Mental State Examination; aDCSI, adapted Diabetes Complications Severity Index; ADL, activity of daily living; IADL, instrumental activity of daily living

The next consideration was the quality of the studies included in the present meta-analysis (Table 2). According to each domain, the rate of appropriate assessment was 100% (8/8) for participant selection, 87.5% (7/8) for confounding variables, 12.5% (3/8) for exposure measurement, 100% (8/8) for blinding of outcome assessors, 12.5% (3/8) for incomplete data, and 100% (8/8) for selective outcome reporting. Bias in an included study often resulted from exposure measurement and incomplete data. Furthermore, because < 10 studies were included, we did not create a funnel plot.

**Table 2:** Risk of bias assessment included in the meta-analysis

No.	Reference	Selection of participants	Confounding variables	Measurement of exposure	Blinding of outcome assessment	Incomplete outcome date	Selective outcome reporting
1	Cacciatore et al. [25]	L	L	L	L	U	L
2	Wang et al. [26]	L	L	L	L	U	L
3	Li et al. [27]	L	L	H	L	U	L
4	Castro-Rodriguez et al. [28]	L	L	L	L	L	L
5	Chode et al. [29]	L	H	H	L	L	L
6	Liccini et al. [30]	L	L	H	L	H	L
7	Chao et al. [31]	L	L	H	L	U	L
8	Li et al. [32]	L	L	H	L	L	L

L, low risk of bias; U, unclear risk of bias; H, high risk of bias

### Mortality

**Fig. 2:**
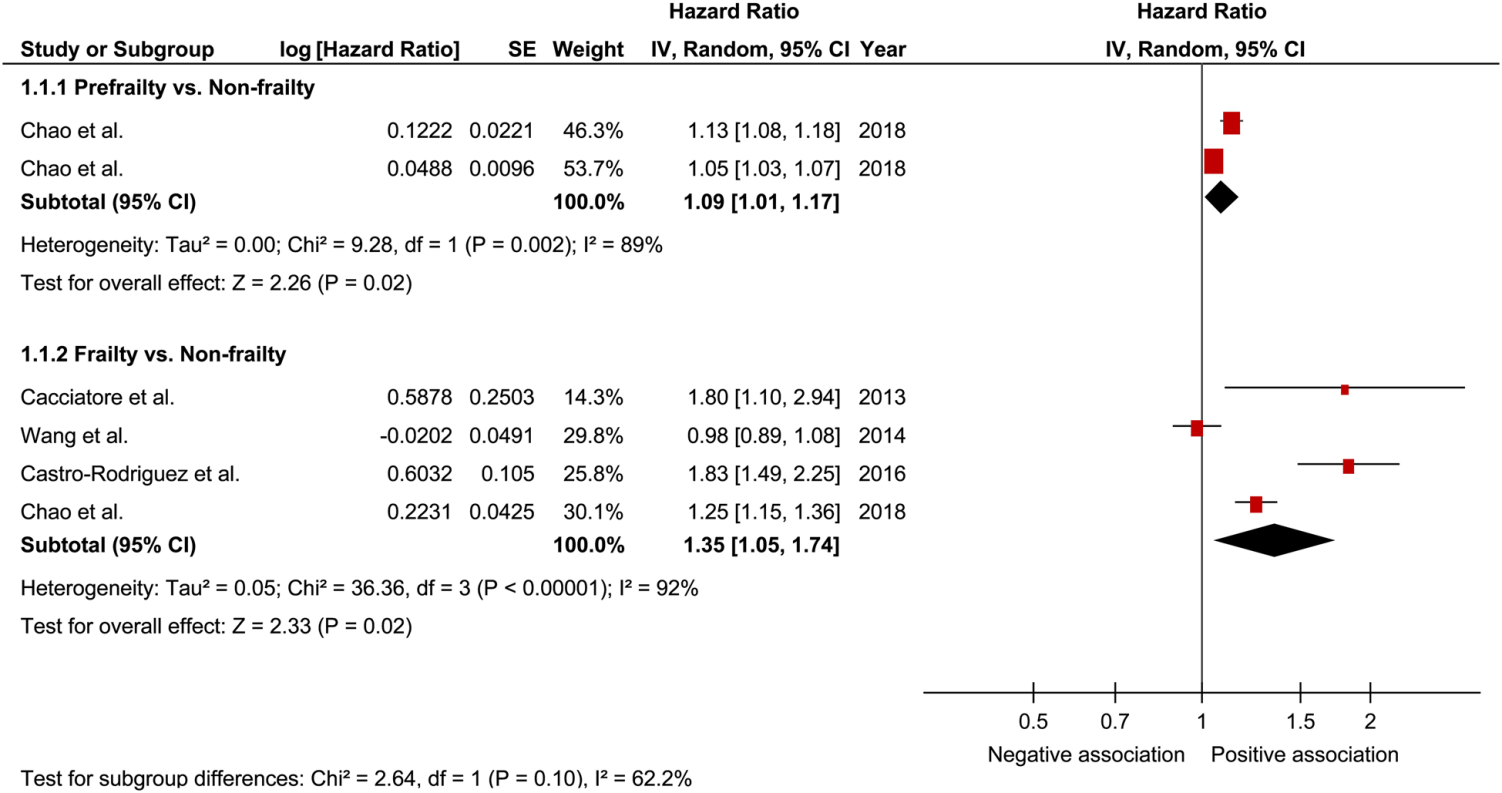
Forest plot of the associations between prefrailty or frailty and mortality. Hazard ratio in the individual studies are presented as squares with 95% confidence intervals (CIs) presented as extending lines. The pooled hazard ratio with its 95% CI is depicted as a diamond

**Fig. 3:**
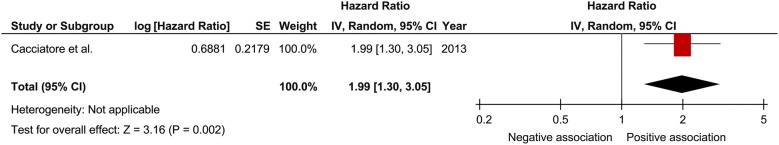
Forest plot of the associations between frailty and mortality in men. Hazard ratio in the individual studies are presented as squares with 95% confidence intervals (CIs) presented as extending lines. The pooled hazard ratio with its 95% CI is depicted as a diamond

**Fig. 4:**
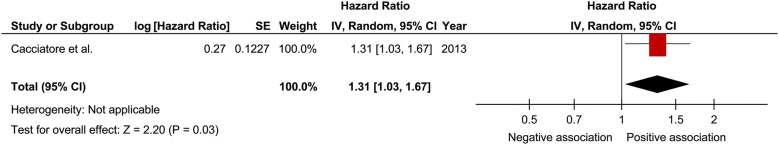
Forest plot of the associations between frailty and mortality in women. Hazard ratio in the individual studies are presented as squares with 95% confidence intervals (CIs) presented as extending lines. The pooled hazard ratio with its 95% CI is depicted as a diamond

**Fig. 5:**
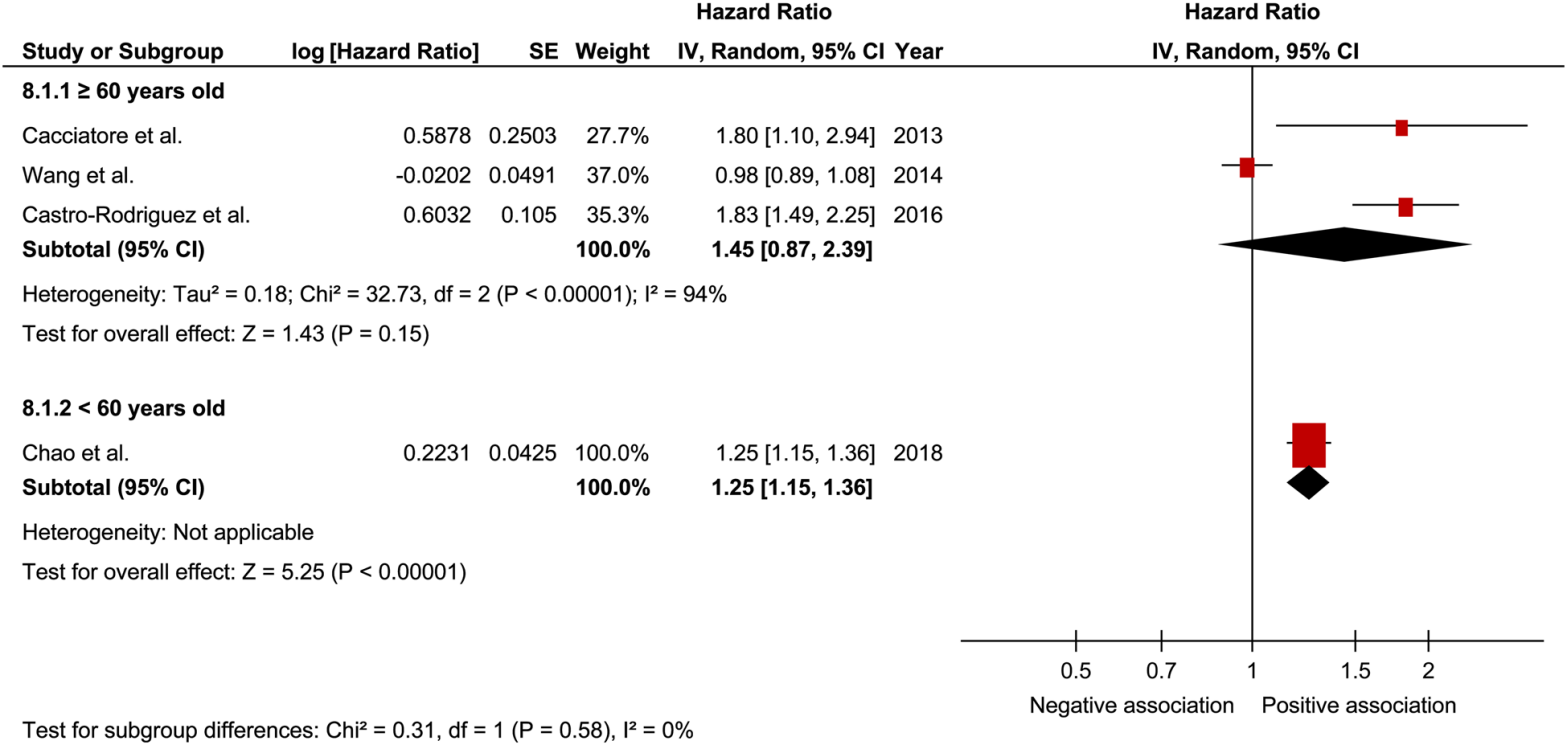
Forest plot of the association between frailty and mortality plotted based on subgroup analysis. Hazard ratio for individual studies is presented as squares, with 95% confidence intervals (CIs) presented as extending lines. Pooled hazard ratio with its 95% CI is depicted as a diamond

**Fig. 6:**
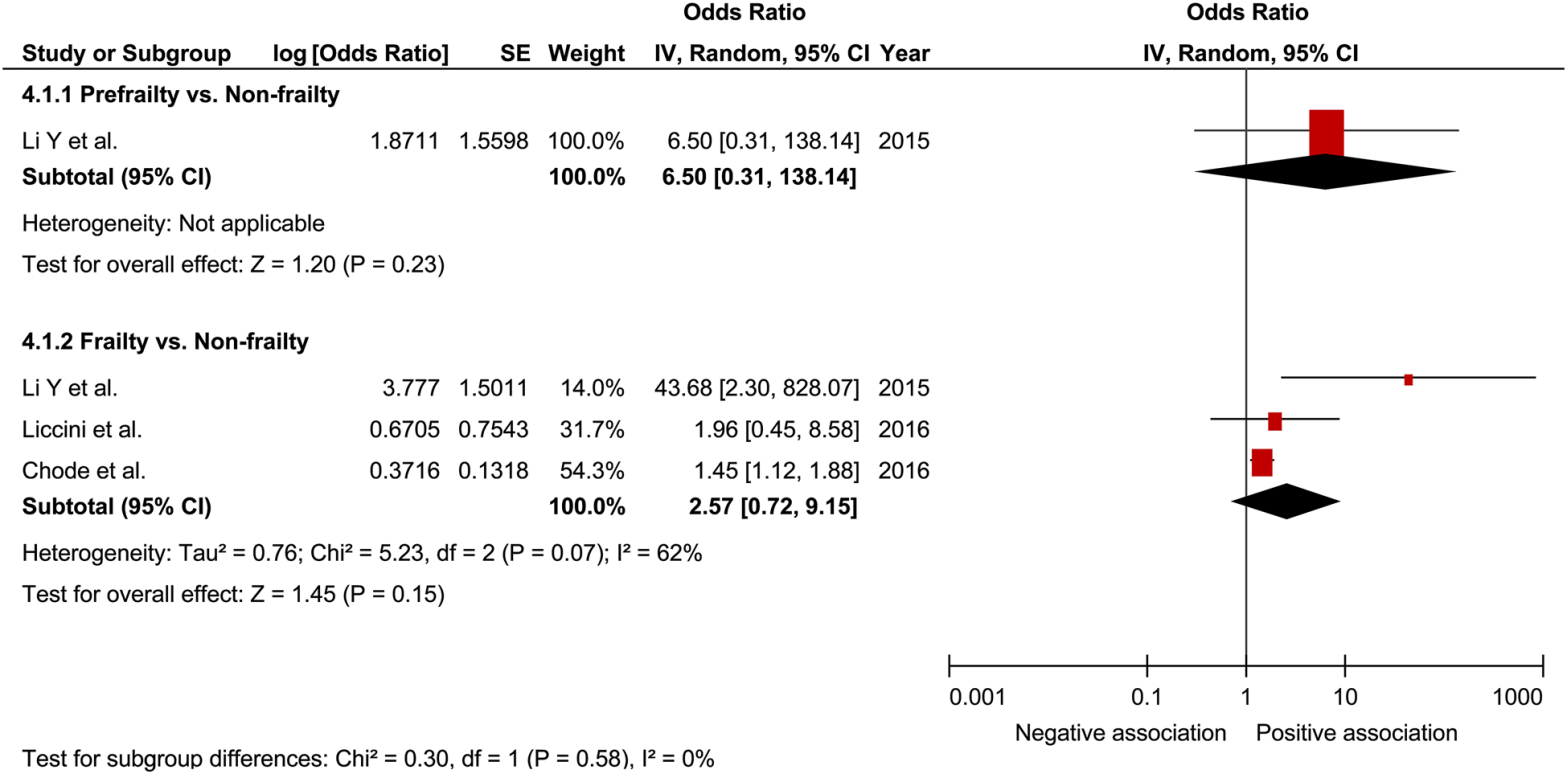
Forest plot of the associations between prefrailty or frailty and mortality. Odds ratio in the individual studies are presented as squares with 95% confidence intervals (CIs) presented as extending lines. The pooled odds ratio with its 95% CI is depicted as a diamond

In a comprehensive analysis involving HR as an effect measure, 4 studies were included [25, 26, 28, 31]. For mortality, the pooled HR of prefrailty was found to be significantly associated with that of frailty (1.09; 95% CI 1.01–1.17; P = 0.02; *I*^2^ = 89% and 1.35; 95% CI 1.05–1.74; P = 0.02; *I*^2^ = 92%, respectively; Fig. 2). In the analysis of the effect of frailty on mortality according to sex, only one study was included for each sex [25]. The pooled HR of prefrailty related to mortality in males and females was 1.99 (95% CI 1.30–3.05; P = 0.002; Fig. 3) and 1.31 (95% CI 1.03–1.67; P = 0.03; Fig. 4), respectively, showing a significant relationship. In a comprehensive analysis with OR as an effect measure, 3 studies were included [27, 29, 30]. Subgroup analysis for age showed that the pooled HR of frailty related to mortality was 1.45 (95% CI 0.87 to 2.39; P = 0.15; Fig. 5) and 1.25 (95% CI 1.15 to 1.36; P < 0.001; Fig. 5) in patients aged ≥ 65 years and those aged < 65 years, respectively. The pooled OR of prefrailty and frailty related to mortality was 6.50 (95% CI 0.31 to 138.14; P = 0.23; Fig. 6) and 2.57 (95% CI 0.72 to 9.15; P = 0.15; *I*^2^ = 62%; Fig. 6), with no significant relationship.

### Hospitalization

**Fig. 7:**
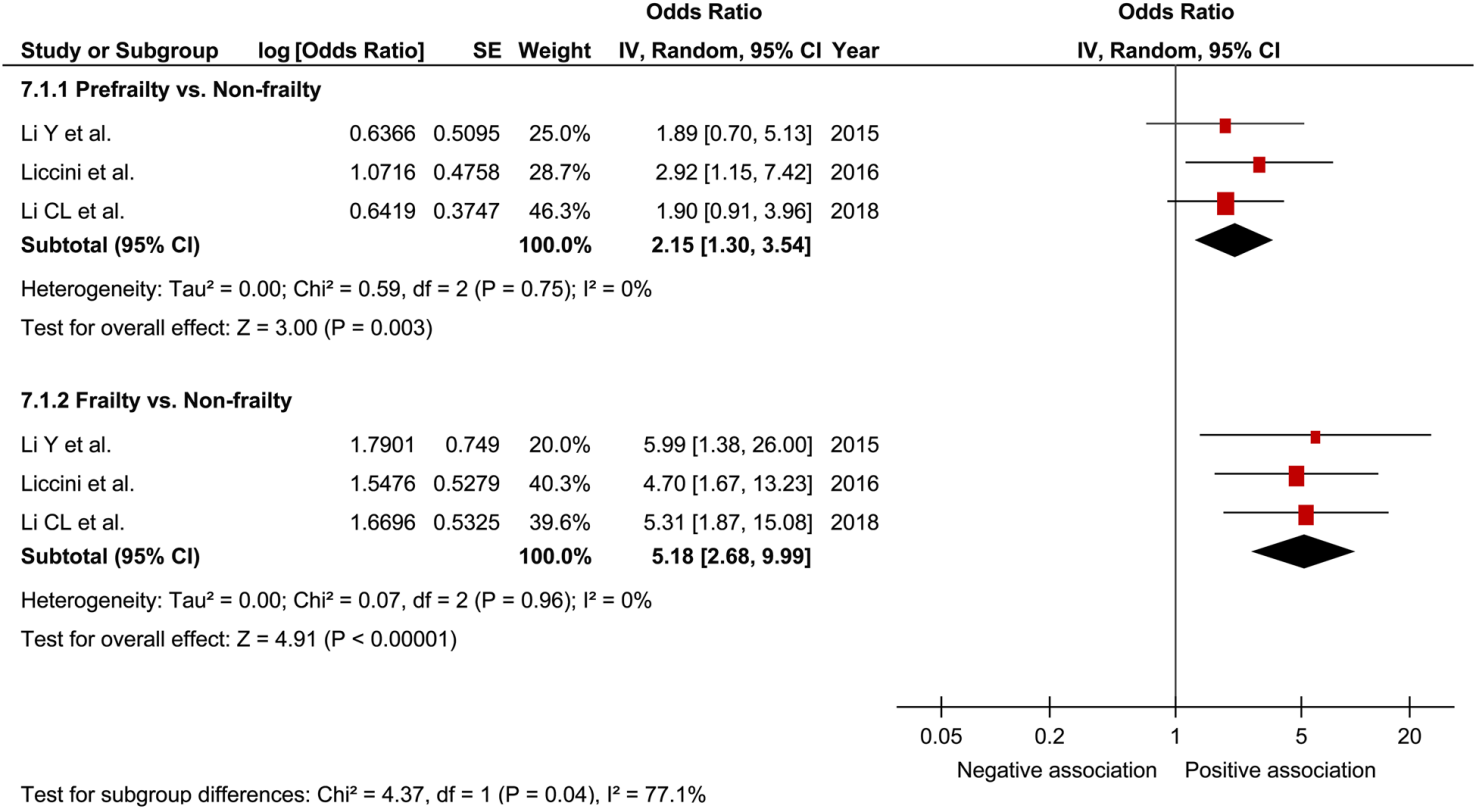
Forest plot of the associations between prefrailty or frailty and hospitalization. Odds ratio in the individual studies are presented as squares with 95% confidence intervals (CIs) presented as extending lines. The pooled odds ratio with its 95% CI is depicted as a diamond

**Fig. 8:**
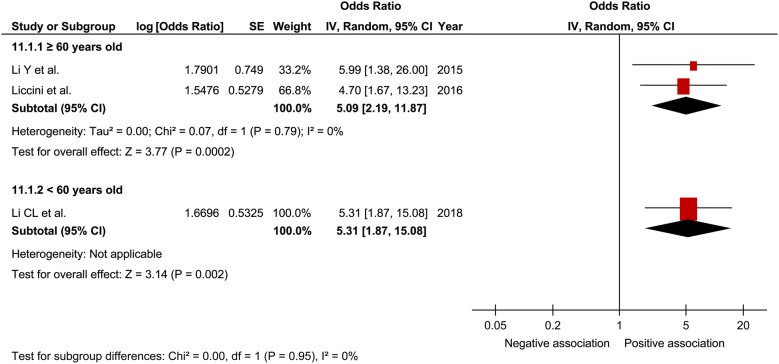
Forest plot of the association between frailty and hospitalization plotted based on subgroup analysis. Odds ratio for individual studies is presented as squares, with 95% confidence intervals presented as extending lines

**Fig. 9:**
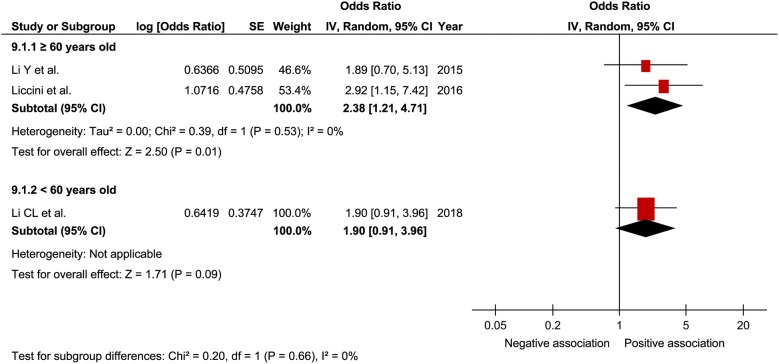
Forest plot of the association between prefrailty and hospitalization plotted based on subgroup analysis. Odds ratio for individual studies is presented as squares, with 95% confidence intervals presented as extending lines

**Fig. 10:**
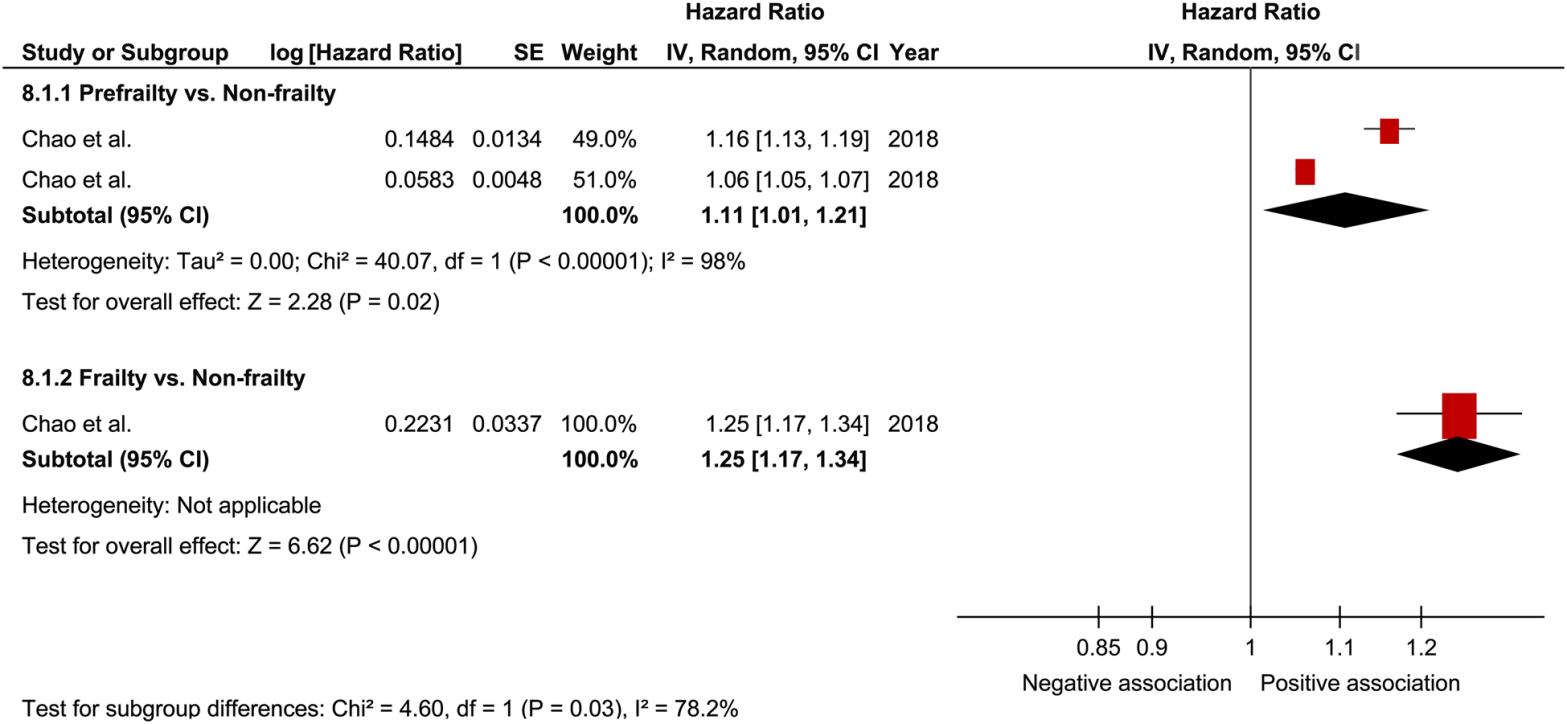
Forest plot of the associations between prefrailty or frailty and hospitalization. Hazard ratio in the individual studies are presented as squares with 95% confidence intervals (CIs) presented as extending lines. The pooled hazard ratio with its 95% CI is depicted as a diamond

In a comprehensive analysis with OR as an effect measure, 3 studies were included [27, 30, 32]. The pooled OR of prefrailty and frailty related to hospitalization was 2.15 (95% CI 1.30–3.54; P = 0.003; *I*^2^ = 0%; Fig. 7) and 5.18 (95% CI 2.68–9.99; P < 0.001; *I*^2^ = 0%; Fig. 7), respectively, with a significant relationship. Subgroup analysis for age showed a significant relationship between frailty and hospitalization in patients aged ≥ 65 years and those aged < 65 years (Fig. 8). In contrast, the pooled OR of prefrailty related to hospitalization was 2.38 (95% CI 1.21 to 4.71; P = 0.01; Fig. 9) and 1.90 (95% CI 0.91 to 3.96; P = 0.09; Fig. 9) in patients aged ≥ 65 years and those aged < 65 years, respectively. In a comprehensive analysis with HR as an effect measure, only 1 study was included [31]. The pooled HR of prefrailty and frailty related to hospitalization was 1.11 (95% CI 1.01–1.21; P = 0.02; *I*^2^ = 98%; Fig. 10) and 1.25 (95% CI 1.17–1.34; P < 0.001; Fig. 10), respectively, with a significant relationship.

### Cardiovascular diseases

**Fig. 11:**
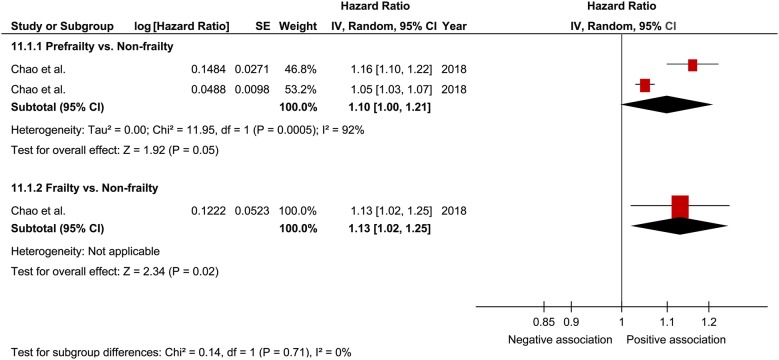
Forest plot of the associations between prefrailty or frailty and cardiovascular disease. Hazard ratio in the individual studies are presented as squares with 95% confidence intervals (CIs) presented as extending lines. The pooled hazard ratio with its 95% CI is depicted as a diamond

Only 1 study was included in the analysis for cardiovascular diseases [31] in which the pooled HR of prefrailty and frailty related to cardiovascular disease was 1.10 (95% CI 1.00–1.21; P = 0.05; *I*^2^ = 92%; Fig. 11) and 1.13 (95% CI 1.02–1.25; P = 0.02; Fig. 11), respectively, with a significant relationship found only for frailty. The limited number of studies prevented the performance of subgroup analysis for the relationship between prefrailty/frailty and cardiovascular diseases.

## Discussion

The present study examined the relationship between frailty and mortality, hospitalization, and cardiovascular diseases in patients with diabetes using a meta-analysis. As a result, prefrailty and frailty were found to have a significant relationship with mortality and hospitalization. Regarding cardiovascular diseases, although a relationship was found with prefrailty and frailty, only 1 study was included, thereby not providing robust results.

According to the meta-analysis of previous studies that examined the relationship between frailty and mortality in community-dwelling individuals, the pooled HR of prefrailty related to mortality was 1.75 (95% CI 1.14–2.70) [33]; when calculated for frailty, the risk of mortality increased by 1.8–2.3-fold [34]. In the present study, while a significant relationship was observed between the pooled HR of prefrailty and frailty related to mortality, we thought that the pooled HR was lower than that reported in previous studies. Among the studies included in our meta-analysis, in the study by Chao et al. [31], the participant sample size was larger than that included in other studies; moreover, the patients were in their 50 s, which is relatively young. The impact of frailty on mortality increases with age [35, 36]; therefore, it is possible that the pooled HR of frailty related to mortality was underestimated in the present study. In contrast, the relationship between frailty and mortality was observed only in patients aged < 60 years in the subgroup analysis. A previous study involving patients with type 2 diabetes indicated that the impact of diabetes on mortality was higher in middle-aged patients than in elderly patients [37]. This might be caused by higher smoking and obesity rates as well as lower prescription rates of statins in middle-aged patients with diabetes than in elderly patients with diabetes [37]. Middle-aged patients with diabetes with an increased risk of death possibly have a huge impact of frailty on their mortality. However, given the small sample size of patients aged ≥ 65 years, the results of the present study may be underpowered. Thus, further studies are required to examine the impact of frailty on mortality according to age. When analyzing the comprehensive relationship of prefrailty and frailty with mortality using pooled ORs, no significant difference was noted. It was inferred that a relationship was not observed because of the small sample size and statistical power.

In a meta-analysis on the relationship between frailty and hospitalization in community-dwelling individuals, the pooled OR of prefrailty and frailty related to hospitalization was 1.26 (95% CI 1.18–1.33) and 1.90 (95% CI 1.74–2.07), respectively [17]. In the present study, the pooled OR of prefrailty and frailty related to hospitalization was higher than that reported in previous studies, thus suggesting that frailty contributes to hospitalization in patients with diabetes. Furthermore, *I*^2^ was 0% in our analysis with no heterogeneity; thus, it was thought that the results were robust to a certain degree. Although the subgroup analysis for age showed a relationship between frailty and hospitalization regardless of age, a relationship between prefrailty and hospitalization was observed only in patients aged ≥ 60 years. Some previous studies [27, 30, 32] have indicated that age possibly strongly influences prefrailty related to hospitalization, consistent with our results. Therefore, caution for hospitalization is clinically important in prefrail elderly patients with diabetes. When analyzing the comprehensive relationship between prefrailty and frailty related to hospitalization using pooled HR, we assumed that HR was relatively small, although a significant relationship was observed between them. It was inferred that the young age of the study participants included in this analysis could have affected the results.

In the present meta-analysis, very few included studies examined the relationship between frailty and cardiovascular diseases; as a result, robust results were not obtained. In previous studies on community-dwelling individuals, it was reported that prefrailty and frailty are the independent risk factors for cardiovascular diseases [38]. In the present meta-analysis, there was only one study [31] that examined the relationship between frailty and cardiovascular diseases in patients with diabetes; therefore, we believe that further analysis using more studies is warranted.

Although the mechanism underlying the relationship of frailty with mortality, hospitalization, and cardiovascular diseases in patients with diabetes remains largely unclear, the following mechanism is considered. Frailty is closely associated with reduced physical and/or cognitive function [39], which leads to poor vital prognosis [40, 41]. It is possible that performing less physical activity along with reduced cognitive function will contribute to the relationship between frailty and prognosis. Furthermore, as another mechanism, it is suggested that hypoglycemia is involved. In previous studies, hypoglycemia has been found to be associated with a risk of increased mortality and cardiovascular diseases [42, 43]. Reportedly, the prevalence of hypoglycemia increases with frailty [44], and hypoglycemia may contribute to the relationship of frailty with mortality and cardiovascular diseases. Furthermore, in the present meta-analysis, a particularly robust relationship was observed between frailty and hospitalization. Previous studies [45, 46] have suggested that accidental falls are involved as the mechanism linking frailty and hospitalization. It is believed that falls are common among patients with diabetes [47], and it is possible that falls contribute even more to the relationship between frailty and hospitalization. In addition, severe hypoglycemia and its complications are closely associated with hospitalization in patients with diabetes [5, 6]. In patients who are frail and have diabetes, the accumulation of factors such as falls, severe hypoglycemia, and its complications may contribute to hospitalization. However, in the present meta-analysis, the reason for hospitalization was not determined; thus, further examination of these mechanisms is needed.

In patients with diabetes, death and cardiovascular diseases are attributed to classical risk factors such as hypertension, dyslipidemia, and smoking in approximately 60% of the patients [14], and the contributing factor for the remaining 40% is frailty [14–16]. A previous study reported that frailty is a prognostic factor for mortality independent of diabetes-related complications [25]. In patients with diabetes, frailty is now considered an important predictor of vital prognosis [48], and the importance of medical care that takes frailty into consideration has been proposed [49]. Nutrition for frailty [19], exercise [20, 21], and avoidance of hypoglycemia [39] may prevent the exacerbation of or improve frailty, and it is thought that early detection of frailty and early intervention are important against frailty. In the future, further examination is warranted to assess the effect of therapeutic intervention on vital prognosis and hospitalization for patients with diabetes and frailty.

The present meta-analysis has several limitations. First, we cannot eliminate the possibility of relevant studies in the databases that we do not use for literature search in our meta-analysis, which may have affected the results. Second, our meta-analysis includes some studies wherein the adjustment for confounding factors is considered inadequate, which may have caused a bias. Third, the definition of frailty used in the included studies differs among the studies, which may have affected the results. Fourth, heterogeneity is particularly high in the analysis involving mortality and cardiovascular diseases as the outcomes, which may have also affected the results. Lastly, relatively few studies were included in our meta-analysis, and a subgroup analysis could not be performed. We believe that re-examination to overcome these limitations is needed in the future wherein more studies can be included.

## Conclusions

The present study used a meta-analysis to examine the relationship between frailty and mortality, hospitalization, and cardiovascular diseases in patients with diabetes. Frailty showed a relationship with mortality and hospitalization. Although a relationship was observed between frailty and cardiovascular diseases, this finding was based on very few studies; thus, robust results could not be obtained. We believe that further examination is needed in the future that considers the aforementioned limitations.

## Data Availability

The data that support the findings of this study are available from the corresponding author upon reasonable request.

## Abbreviations

HR: hazards ratio
OR: odds ratio
CI: confidence interval
QOL: quality of life
